# Proteome mapping of *Plasmodium*: identification of the *P. yoelii* remodellome

**DOI:** 10.1038/srep31055

**Published:** 2016-08-09

**Authors:** Anthony Siau, Ximei Huang, Mei Weng, Siu Kwan Sze, Peter R. Preiser

**Affiliations:** 1 Nanyang Technological University, School of Biological Sciences, 637551, Singapore

## Abstract

*Plasmodium* associated virulence in the host is linked to extensive remodelling of the host erythrocyte by parasite proteins that form the “remodellome”. However, without a common motif or structure available to identify these proteins, little is known about the proteins that are destined to reside in the parasite periphery, the host-cell cytoplasm and/or the erythrocyte membrane. Here, the subcellular fractionation of erythrocytic *P. yoelii* at trophozoite and schizont stage along with label-free quantitative LC-MS/MS analysis of the whole proteome, revealed a proteome of 1335 proteins. Differential analysis of the relative abundance of these proteins across the subcellular compartments allowed us to map their locations, independently of their predicted features. These results, along with literature data and *in vivo* validation of 61 proteins enabled the identification of a remodellome of 184 proteins. This approach identified a significant number of conserved remodelling proteins across *plasmodium* that likely represent key conserved functions in the parasite and provides new insights into parasite evolution and biology.

After invasion into the erythrocyte, the malaria parasite is found within a parasitophorous-vacuole (PV) enclosed by a PV membrane (PVM). Here, *Plasmodium* extensively modifies the host erythrocyte by secreting a range of proteins beyond the parasite plasma membrane (PPM). *Plasmodium* proteins are found in the PV, the PVM, the host-cell cytoplasm (HCC) and the erythrocyte membrane (EM). In the HCC, proteins are found uniformly distributed as soluble proteins and/or localized in specialized structures that are so far poorly characterized. Despite their critical role in parasite survival, the parasite proteins exported beyond the plasma membrane to remodel the host-cell remain poorly characterized. This set of proteins that we named “remodellome” includes not only the set of molecules exported beyond the PVM and commonly named “exportome”, but also the proteins exported to the host parasite interface.

The screening of the parasite proteome/genome for proteins that fulfils the discriminative criteria associated with remodelling proteins identified a unique PEXEL/VTS motif that targets a repertoire of ~300–400 exported proteins in *Plasmodium falciparum*^1^,^2^,^3^,^4^. These proteins are translocated across the PVM by a protein cargo complex named PTEX, proposed using predictive criteria^5^. However, the increasing number of PEXEL-Negative Exported Proteins (PNEP) indicated that the predicted PEXEL/VTS exportome is not inclusive of all exported proteins^6^. In absence of a conserved signature sequence, it is currently not possible to predict the whole extent of the PNEP exportome. The identification of PNEP still depends on time consuming approaches using general criteria such as gene expression profile and genomic localization^7^. Approximately 75% of the *P. falciparum* exportome currently known is a result of the expansion of PEXEL protein families, leaving an exportome of ~100–150 PEXEL and non-PEXEL proteins mostly unique to this parasite^3^. In other *Plasmodium* species, the small number of predicted PEXEL proteins identifiable supports that PNEP could play a more prominent role^6^. A recent study focusing on the export requirement of PNEP in *P. yoelii* identified a novel export motif named PLASMED that was used to predict an exportome of ~670 proteins, mostly encoded by genes belonging to multigene families like the PIR (YIR in *P. yoelii,* conserved in all the genus at the exception of *P. falciparum*) and the PYST family (conserved in all the rodent parasite)^8^.

To overcome the apparent limitations of focusing on discriminative protein features, an alternative approach based on the systemic proteomic analysis of parasite samples enriched for specific subcellular compartments was used. Analysis of parasite samples enriched in PV^9^, infected EM^10^,^11^,^12^ or parasite-induced HCC structure proteins^13^,^14^ identified expanded sets of remodelling proteins that included contaminating intracellular proteins. These datasets were subsequently refined to subtract contaminating proteins using strategies that likely would also remove proteins of interest^10^,^11^,^12^,^13^. Here, we developed a strategy to determine the *P. yoelii* remodellome. For this, RBC infected by *P. yoelii* were fractionated in order to separate the parasite remodellome from those of the internal parasite. Differential analysis of the protein abundance between fractions enriched in remodelling proteins and those including the intraerythrocytic parasite allowed us to distinguish the parasite remodellome, independently of the protein’s features. This allowed us not only to validate the cellular location of previously identified PEXEL, PLASMED and PNEP proteins, but also to identify a range of new abundant PNEP. Many of these PNEP are conserved across different *plasmodium* species suggesting that they play a critical and conserved role in parasite host-cell remodelling.

## Results

### Subcellular fractionation of *P. yoelii* infected erythrocyte

**Figure 1:**
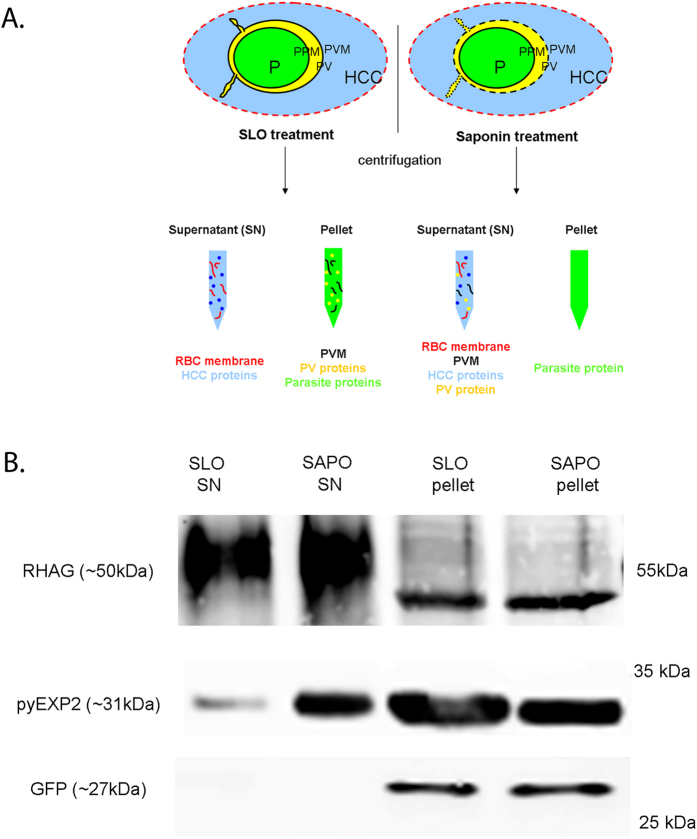
Subcellular fraction of *P. yoelii* infected RBC. (**A**) Schematic representation of the subcellular fractionation workflow used to map the whole *P. yoelii* exportome. P: parasite, PV: parasitophorous vacuole, PPM: parasite plasma membrane, PVM: parasitophorous vacuole membrane, HCC: host-cell cytoplasm. Parasites expressing non-exported GFP chimera were treated with SLO or Saponin and then centrifuged to obtain a supernatant and a pellet fraction. The lysed RBC membrane is represented by a red dotted line. The composition of each SN/pellet sample obtained is indicated in red. (**B**) Assessment of the PVM, PPM and RBC membrane integrity following selective permeabilization using SLO and Saponin. *P. yoelii* infected RBC expressing a non-exported GFP tag were treated with SLO or Saponin (SAPO) and centrifuged to separate the supernatant (SN) from the pellet. Antibodies against the PVM marker EXP2 (pyEXP2), the intraerythrocytic GFP were used to evaluate the integrity of the PVM and PPM, respectively. The antibody against Rhesus blood group-associated glycoprotein (RHAG) was used to evaluate the integrity of the RBC membrane. Each lane was loaded with ~0.5 μl (pyEXP2 and GFP) or ~1 μl (RHAG) of equivalent parasites. The antibodies used and their cognate expected molecular weight is indicated on the left. The molecular weights on the right indicate the positions to which size markers had migrated.

*P. yoelii* infected erythrocyte samples were fractionated to separate proteins translocated to the periphery (peripheral) from those exported to the host-cell (exported) or those remaining in the internal parasite (internal) (Fig. 1A). Purified mature trophozoite/early-schizont parasite cultures expressing a non-exported GFP-tag were divided and treated with either Streptolysin O (SLO) or Saponin (SAPO). SLO permeabilizes the erythrocyte membrane without affecting the PVM, whereas Saponin disrupts the erythrocyte membrane and the PVM without affecting the PPM. The treated cultures were then centrifuged at low speed in order to separate the parasites (pellet) from the HCC content (SN), generating four samples enriched in specific subcellular compartments. They include (1) a SLO SN sample associated with erythrocyte membrane and HCC proteins, (2) a SAPO SN sample enriched in HCC, parasite periphery (PVM and PV) and erythrocyte membrane proteins, (3) a SLO pellet sample enriched the proteins localized in the intraerythrocytic parasite and the parasite periphery and (4) a SAPO pellet sample containing mostly the intraerythrocytic parasite molecules only.

Following treatments, the integrity of the PPM, PVM and the RBC membrane were assessed by western-blot probed with antibodies against GFP, the PVM protein EXP2 9 and the RBC membrane protein Rhesus blood group-associated glycoprotein (RHAG) (Fig. 1B). The non-exported GFP (~27 kDa) was only detected in the SLO and SAPO pellets, indicating that the integrity of the PPM was largely preserved by these treatments. Conversely, anti-EXP2 antibody revealed a ~30 kDa polypeptide in all the fractions. However, the comparatively small amount of EXP2 found in the SLO SN sample indicated that, while this sample was not devoid of cross-contamination, the PVM remained mostly intact during SLO treatment. Finally, the large amount of RHAG protein detected in the supernatants fractions indicated that SLO and saponin treatments efficiently lysed the RBC membrane and that these fractions are enriched in RBC membrane proteins.

### Proteomic profiling of fractionated *P. yoelii* parasite

To map *P. yoelii* sub-proteomes, the sub-cellular samples derived from four independent fractionation experiments were analysed by liquid chromatography coupled tandem mass-spectrometry (LC-MS/MS). Compiling peptide hits from the four subcellular fractionation samples and retaining proteins which can be identified in at least two experiments, 1335 parasite proteins were identified in the four fractions and their abundance calculated using the emPAI index (Supplementary Table S1)^15^,^16^,^17^. The median emPAI value of these proteins was ~0.5 with emPAI value ranging from ~0.01 to ~2800, indicating protein abundance spans four orders of magnitude.

**Figure 2:**
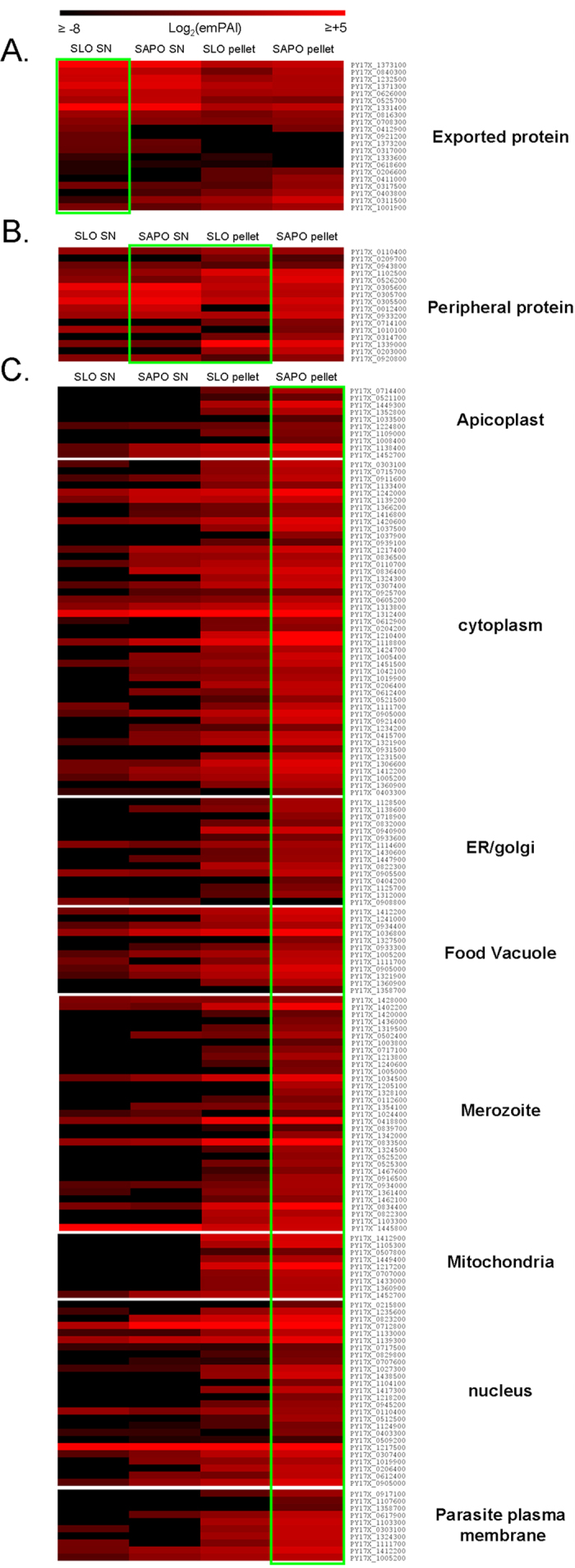
Relative abundance pattern of plasmodial proteins based on their subcellular localization. Of the 1335 proteins identified in infected RBC, the localization of 181 proteins had been characterized in the literature either in the endogenous parasite or as orthologues in other *Plasmodium spp.* These proteins were grouped based on their predictable localization: exported beyond the PVM (**A**), localized in the periphery (**B**) and internal (**C**). The internal proteins were further subdivided based on their intraerythrocytic sub cellular localization: apicoplast, cytoplasm, ER/golgi, food vacuole, forming merozoite, mitochondria, nucleus and PPM. All the protein abundance levels measured using the emPAI index were normalized to the means of emPAI value measured in the supernatant and pellet fractions obtained from the same culture sample. The abundance of each protein in the four fractions analyzed are detailed in the heat map graphically represented using a gradient of red. The magnitude of abundance level of expression is indicated above the figure, from black to red. The green rectangles highlight the cognate subcellular samples mostly associated to exported (SLO SN), peripheral (SAPO SN and SLO pellet) and internal proteins (SAPO pellet). Each protein is represented horizontally, and each subcellular fraction (SLO SN, SAPO SN, SLO pellet and SPO pellet) is represented vertically.

As the sub-proteomes identified here contain proteins previously characterized either in the endogenous parasite or as orthologues in other *Plasmodium spp.*, we searched the literature for proteins with experimental evidences pertaining to their subcellular locations. Overall, 181 proteins (~14% of the proteome) with clear localization data in asexual blood stage parasite, were found (Fig. 2 and Supplementary Table S1) including 16 peripheral proteins, such as the PTEX components EXP2 and PTEX88 5,9,18. Reflecting the low proportion of *P. falciparum* exported protein shared across the genus and the low number of exported proteins with orthologues in other *plasmodium spp.* currently known, 26 exported proteins were identified including the previously characterized PYST proteins^8^,^12^. Finally, 139 proteins were located within the internal parasite including the apicoplast, food vacuole, mitochondrion, cytoplasm, nucleus, forming merozoite, parasite plasma membrane and ER/golgi.

Combining the relative abundance of the annotated proteins together with their localization information, we identified three distinct patterns (Fig. 2). A higher abundance in the SAPO SN or in the SLO pellet fraction, which includes the PV/PVM content, was observed for ten out of 16 peripheral proteins (Fig. 2B). A higher abundance in the SLO SN fraction was found for 16 out of 26 exported proteins (Fig. 2A) while 137 out of 139 internal proteins showed a higher abundance in the SAPO pellet fraction (Fig. 2C). This suggests that proteins associated to the Saponin SN or the SLO pellet were likely peripheral; those mostly found in the SLO SN were exported while those associated with the Saponin pellet fraction were internal.

The localization of the remaining proteome was assigned based on their relative abundance patterns (Supplementary Table S1). For this, putative peripheral proteins characterized by a higher abundance in the Saponin SN and/or the SLO pellet fraction were identified by comparing their emPAI values in these fractions versus those found in the SLO SN and Saponin pellet. Only proteins with a ratio > 1.1 (i.e. with abundance higher by at least 10% in the Saponin SN and/or the SLO pellet fraction as compared to the remaining fractions) were retained. Following this first search, we compared the protein abundances found in the SLO SN fraction to those in the Saponin pellet fraction. The proteins with an emPAI ratio SLO SN/Saponin pellet >0.6 were proposed as exported, whereas the remaining (ratio < 0.6) were considered as internal. The localization predictions obtained using these criterions were combined with those previously obtained from the literature (Supplementary Table S1).

### Validation of the localization predictions

To validate our approach, we assessed the subcellular localization of 61 proteins predicted to be peripheral (n = 9), exported (n = 28) or internal (n = 24) using GFP-tagged proteins. We prioritized uncharacterized proteins encoded by genes ≤2 kb to facilitate the cloning and the episomal expression in *P. yoelii* parasites. The peripheral candidates included a nuclease (PY17X_1033000), six PYST and two YIR proteins. For the exported subset, we took account of the prevalence of PYST and selected 19 PYST annotated as fam-a (n = 16), fam-b (n = 1) and fam-d (n = 2). In addition, we also included a YIR protein (PY17X_0102100), a PEXEL-positive protein (PY17X_0216200), a tryptophan rich antigen (PY17X_0626300), a “zinc finger protein” (PY17X_1214600), the MSP-9 protein PY17X_1445800, two proteins with unknown function (PY17X_1147600 and PY17X_1350000) and two proteins annotated as “CCAT-binding transcription factor-like protein” (PY17X_0520300 and PY17X_0520200). Finally, the 24 proteins predicted to be internal include mostly housekeeping proteins involved in the parasite metabolism, translation and signalling pathways, as well as two proteins with unknown function (PY17X_0621300 and PY17X_1238600).

**Figure 3:**
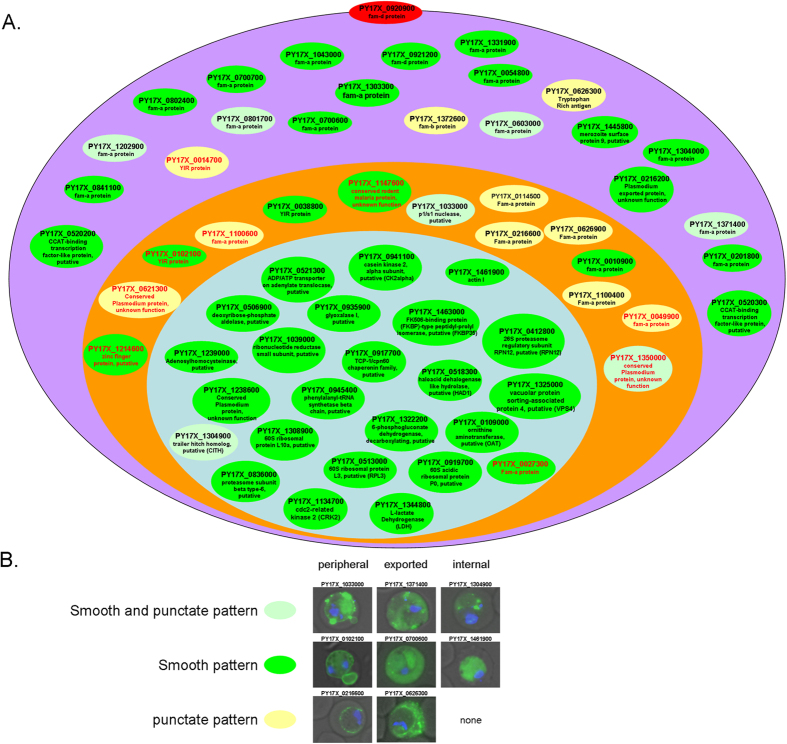
Synopsis of subcellular localization of 61 proteins determined using GFP-tagging. (**A**) The localization of each protein is represented in a schematic infected erythrocyte: either in the HCC cytoplasm (violet), periphery (orange) or the internal parasite (light blue). Each protein is represented by an oval, which provide information about the protein ID, the description available in PlasmoDB database and the fluorescence patterns observed. Overall, three fluorescence patterns can be distinguished. The chimera that displayed a smooth fluorescence pattern in the periphery (n = 5), the HCC (n = 15) and the internal parasite (n = 23) were highlighted in green (Figures S1a, S2a and S3a). Those, that showed a smooth fluorescence pattern with one or several reinforcements in the periphery (n = 2), the HCC (n = 4) or the internal parasite (n = 1) were highlighted in light green (Figures S1b, S2b and S3b). Finally, the chimera that induced a granular pattern in the periphery (n = 7) and the HCC (n = 3), compatible with localization in specialized structures, were highlighted in yellow (Figures S1c and S2c). Finally, PYST PY17X_0920900 chimera displayed a fluorescence pattern associated with the RBC membrane (Figure S4) and was represented by a red oval in the RBC membrane. The nine proteins mispredicted are highlighted in red. (**B**) Representative merged image of transfectants displaying the three GFP fluorescence patterns observed. The type of GFP pattern observed is represented horizontally and each group of subcellular localization (peripheral, exported or internal) is represented vertically. The name of the GFP chimera is indicated above the picture. None of the internal proteins screened displayed a punctate pattern.

Localization of the 61 GFP- tagged chimeras was ascertained using live-cell fluorescence microscopy performed on mixed-stage blood stage transfectants stained with DAPI. This allowed us to discern the nucleus, as well as the internal structures and membranes of live parasites, including the PVM and the RBC membrane^8^. Observation of the transfectants revealed three main fluorescence patterns (Fig. 3 and Figures S1–S3) and indicated that the localization of 52 proteins (~85% accuracy) were correctly predicted. It includes seven (out of the 9) putative peripheral proteins, 22 (out of the 28) exported candidates including the PYST PY17X_0920900 that localized with the RBC membrane (Figure S4), and 23 (out of the 24) proteins predicted as internal (Fig. 3 and Figures S1–S3). Nine proteins were incorrectly predicted including an exported and an internal protein mispredicted as peripheral (Figures S2 and S3 and highlighted in red in the Supplementary Table S1) and seven peripheral proteins mispredicted as exported (n = 6) or internal (n = 1) (Figure S1). The reasons behind these discrepancies remain to be individually determined. It could be linked to the partial alteration of the PVM integrity noted Fig. 1 that could release contaminant proteins in the SLO SN fraction or the limitations inherent to the expression of proteins fused to a large fluorescent reporter from episomal constructs using the strong constitutive promoter *eef1*-α. Hence, the proteome map presented in Supplementary Table S1 was updated to reflect the localization evidence by favoring (1) localization data obtained from homologous individual validation (*P. yoelii*, this study and literature data), (2) heterologous information (literature data) and lastly 3) proteomic prediction. It includes 52 peripheral, 132 exported and 1151 internal proteins.

### Analysis of the *P. yoelii* proteome

**Figure 4:**
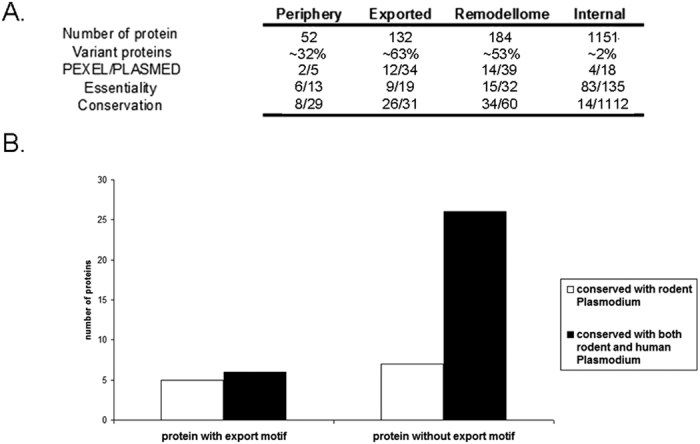
Analysis of the *P. yoelii* remodellome. (**A**) Fact sheet of the proteins identified by proteomic analysis and classified into peripheral (n = 52), exported (n = 132) and internal (n = 1151) proteins subset following, GFP-tagging, literature data or relative abundance pattern. The remodellome (n = 184) columns summarize the information collected from both the peripheral and the exported subsets. Variant proteins indicate the proportion of variant proteins (PYST, YIR and PY235 families) within each of these subsets. PEXEL/PLASMED depicts the number of PEXEL and PLASMED motif found. Essentiality shows for each subset, the number of protein with syntenic orthologue in *P. falciparum* or *P. berghei* shown to be essential (no gene-deleted parasite obtainable) or requisite (gene-deleted parasites with a growth defect) for the parasite growth out of the total number of proteins with reverse genetic data found. Conservation indicates the number of non-variant proteins conserved exclusively amongst rodent parasites along with those conserved across both human and rodent *Plasmodium*. (**B**) Exported proteins with PLASMED/PEXEL motifs are less conserved than those without export motif. Number of non-variant exported protein with or without PEXEL/PLASMED that are conserved amongst rodent surrogate or both human and rodent *Plasmodium*. While only 5 out of 11 PEXEL/PLASMED proteins are shared across the genus, this proportion is 27 proteins out of 33 for PEXEL/PLASMED negative proteins.

The *P. yoelii* remodellome is characterized by a large number of variant proteins, supporting the existence of remodelling features specific to rodent parasites (PYST) or common to all the parasite species at the exception of *P. falciparum* (YIR) (Fig. 4 and Supplementary Table S1). The predominance of variant proteins in the periphery, with 14 PYST and three YIR predicted (~32% of the peripheral subset, Fig. 4A) was supported by the localization pattern observed with seven PYST and two YIR (Fig. 3 and Figure S1). Variant proteins also make up ~63% of the exportome, suggesting that they play a critical role in the HCC (Fig. 4a and Supplementary Table S1). PYST proteins form the largest and the most abundant group of exported candidates, with 66 members annotated as fam-a (n = 57), fam-b (n = 7) and fam-d (n = 2). Thirty-four PYST are detected with an emPAI value superior to the median emPAI (0.5). The second largest group of exported proteins is the YIR, with 19 members detected with a lower abundance (emPAI value ranging from 0.13 to 0.29 in the SLO SN fraction). The exportome also include three proteins belonging to the PY235 family known to be involved in merozoite invasion (PY17X_0501400, PY17X_1249900 and PY17X_1302700)^19^. Although the presence of these proteins in the HCC needs to be further confirmed, rhoptry proteins have been shown to be transferred to the ring PVM upon invasion (for review see)^20^, suggesting that PY235 proteins are *bona fide* remodelling proteins.

The 79 remaining remodelling proteins include 26 proteins with unknown function and 53 proteins with annotations in the databases. Except for the orthologues of the PTEX translocon components involved in the export of protein beyond the PVM^5^, the role of these proteins in host-remodelling remains elusive. Out of these proteins, 13 peripheral and 19 exported proteins have syntenic orthologues that were previously assayed using gene deletion approaches (Supplementary Table S1). For each of these subsets, less than half of the proteins were shown to be essential or requisite for parasite development, suggesting a noteworthy level of functional redundancy (Fig. 4A, Supplementary Table S1). However, the fact that most of these proteins are shared with human and rodent *Plasmodium* (n = 60/79), strongly supports the existence of remodelling features common to all parasite species (Fig. 4A). The remaining proteins are conserved amongst rodent parasites arguing the existence of lineage-specific remodelling features involving non-variant proteins along with those involving PYST proteins.

Altogether, the analysis of the conservation pattern of the remodellome indicated three levels of remodelling functions: (1) those specific to rodent parasites and mediated by PYST and non-variant proteins, (2) those shared by all the non-*falciparum spp.* and implying YIR and (3) those conserved across the genus and using a core remodellome.

Analysis of the proteome map indicates that the proteins with PEXEL (n = 18) or PLASMED (n = 57) motifs are found predominately in the exportome (12 and 34 exported proteins with PEXEL or PLASMED motifs, respectively) (Fig. 4A and Supplementary Table S1). In line with the previous hypothesis which suggest that non-PEXEL proteins could have a more prominent role in *non-falciparum* species^6^, PLASMED containing proteins make up about 25% of the total exportome while only 9% of the exportome consists of PEXEL proteins. This indicates that PEXEL/PLASMED represent only two possible export motifs and that so far uncharacterized motifs exist to drive the translocation of proteins. Overall, 50% (n = 6/12) and ~79% (n = 27/34) of the PEXEL and PLASMED motifs found in the exportome are associated with variant proteins. PEXEL motifs are exclusively located in PYST proteins while PLASMED motifs are associated with YIR, as shown by the higher proportion of YIR with PLASMED motif (8 proteins out of 19 YIR) compared to this of PYST (19 PLASMED proteins out of 66 PYST). Finally, analysis of the non-variant exportome indicated 11 proteins with a PEXEL/PLASMED motif. Five of these proteins (45%) are conserved across human and rodent *Plasmodium* species while the remaining proteins are rodent parasite specific (Fig. 4B). This contrasts with the non-variant proteins exported without PEXEL/PLASMED motif, for which >80% are conserved across the genus (26 proteins out of 33, Fig. 4B), suggesting that PEXEL/PLASMED proteins expanded during evolution after speciation, to facilitate parasite specific features.

Finally, the 1151 internal proteins are mostly involved in housekeeping functions such as metabolic processes, nucleic metabolism, ubiquitin-signaling pathway, proteolytic and signalization pathways, transportation, translocation machinery, cytoskeleton, stress response, protein synthesis. These proteins are highly conserved across multiple *Plasmodium* species with, 96% of the internal proteins (n = 1112) shared between rodent and human parasites (Fig. 4A and Supplementary Table S1). Unlike remodelling proteins, internal proteins are mostly required for parasite development, with 71 essential, 12 requisite and 52 dispensable proteins found out of 135 proteins with syntenic orthologues previously assayed using reverse genetic approach (Fig. 4A, Supplementary Table S1). In addition, six proteins showed a different phenotype between human and rodent *Plasmodium,* possibly due to differences in the experimental conditions or in the nature of the mutation induced. Overall, these results indicate that internal proteins encode a high number of housekeeping features required for parasite development.

## Discussion

In summary, this study reports the mapping of the *Plasmodium* proteome. The proteome map, validated by accessing the localization of 61 proteins using GFP-tagging, provides a detailed overview of the remodellome during trophozoite/early schizont stages and serves as reference point to understand parasite host-cell remodelling. In addition to exported proteins, the differential analysis of the fractionated samples obtained from SLO and Saponin treatment allowed us to predict the proteins localized in the parasite periphery. However, due to the limitations inherent to the GFP-tagging method, which did not allow us to differentiate the PV/PVM proteins from those localized in the PPM, the peripheral subset is enriched in proteins localized at the host-cell interface, most likely in the PV/PVM. Of interest, 78% of remodelling candidates characterized in our study had abundance profiles correlated with their GFP-tagging localization data (seven out of nine peripheral proteins and 22 out of 28 exported proteins). This proportion was somehow higher than the 62% correlation noted for the remodelling proteins found in the literature (ten out of 16 peripheral proteins, 16 out of 26 exported proteins) and could be due to the heterologous origin of most of the literature data, species-specific difference as well as the limited sample size. While the number of proteins tested needs to be further expanded to draw definitive conclusion, the accuracy rate was not biased by the large proportion of variant proteins accessed. Out of 37 peripheral and exported candidates tested, 28 were variant and nine were non-variant proteins. Following GFP-tagging validation, eight (out of 37) proteins showed a localization not in line with their abundance profile including five variants proteins (error rate of 5/28 = ~18%) and three non-variant proteins (error rate of 3/9 = 33%). Importantly, the approach developed here allowed us to differentially distinguish internal protein with a high reliability (23 internal proteins correctly predicted out of 24 tested). This was further supported by analyzing the abundance profile of internal proteins found in the literature with 137 proteins out of 139 displaying a cognate abundance profile. Altogether, the ~85% of correlation noted between our whole proteome map and GFP-tagging data is comparable to the 90% of correlation noted between literature data and proteome map (ten out of 16 for peripheral proteins, 16 out of 26 for exported proteins and 137 out of 139 for internal proteins).

While the high-throughput proteomic approach used here is able to assess the complexity of the whole malaria proteome, it is important to note that the limits of detection of the approach as well as other technical limitations might compromise the detection of some proteins. To constitute the *P. yoelii* proteome presented in the Supplementary Table S1, we privileged first homologous localization data when available and secondly heterologous data from other *Plasmodium* species. Finally, in the absence of individual validation data, the localization of the proteins identified by proteomic analysis were assigned manually based on the three subsets of subcellular fractionation profiles that were initially defined using literature localization data. With 53% of the remodellome, variant proteins form the largest group of remodelling proteins. Despite this predominance, it may be still underestimated as PIR with >800 members in *P. yoelii* (and to some extend PYST with >210 members), exhibit a high degree of conservation that could decrease the number of unique peptides available for identification and abundance calculation. Supporting the prevalence of variant proteins in the remodellome, variant proteins were found in lipid rafts potentially involved in the assembly and sorting of parasite protein^14^ and constitute a large part of the rodent parasite exportome^8^. The large number of exported variant proteins suggests a critical role in parasite-host interaction and possibly virulence as seen for the *P. falciparum* PfEMP1 family. However, evidence that these proteins are targeted to the erythrocyte surface remains elusive^12^,^21^,^22^,^23^. Among >20 variant proteins screened here, only the PYST PY17X_0920900 appears to be associated with the erythrocyte membrane (Figure S4). All the other variant proteins were apparently targeted to the periphery or the HCC, in soluble form or associated to specialized structures, suggesting that they could have multiple roles within the RBC premise (Figures S1 and S2). Deletion of *P. berghei* PYST proteins had in most cases little impact on parasite growth^12^, suggesting that they are involved directly or indirectly in adaptive remodelling properties that require a large repertoire of functionally redundant proteins. Importantly, the expansion of exported protein families is a feature shared across the genus. While *P. falciparum* protein families are mostly unique to this specie, the conservation pattern of PYST and PIR support the co-existence of remodelling features common to rodent parasite in the case of PYST and rodent, simian and human malaria parasites in the case of PIR. In *P. falciparum,* the absence of PYST and YIR along with the expansion of PEXEL protein families phylogenetically unrelated to rodent parasite, indicate the presence of distinct remodelling features. However, considering that *P. vivax* share PIR with rodent parasites and some cluster of PEXEL protein families with *P. falciparum*^3^, these remodelling features are not exclusively lineage specific and may be shared with other *Plasmodium*. In addition to variant subsets, the presence of a core remodellome characterized by a notable level of functional redundancy suggest that highly conserved remodelling functions are achieved using several alternative pathways. Altogether, the exported patterns noted with the two “CCAT-binding transcription factor-like” proteins (Figure S2), the peripheral pattern observed with the “p1/s1 nuclease” chimera (Figure S1) and the dual localization previously reported for the Histone H3 24, support the existence of remodelling features involving nucleoprotein complexes. This is in line with results indicating that *Plasmodium* can transfer DNA using exosome-like vesicles that participate to quorum-sensing-like mechanisms on a population level along with immunomodulatory properties^25^,^26^,^27^.

Overall, our approach identified two groups of remodelling proteins that have radiated to differing extents, providing insights on the host parasite-interactions. The expansion of protein families differentially conserved across the genus is likely to provide multiple remodelling alternatives allowing the parasite to adapt to the host variability. The conservation of unique proteins through evolution strongly supports the existence of remodelling mechanisms that are functionally essential for the entire genus and are potential targets for the development of new drugs. To date, most of our knowledge about remodelling are biased towards *P. falciparum* which likely has unique remodelling features, as shown by the low proportion of conservation of *P. falciparum* exported proteins across the genus^3^,^4^. The data generated here using *P. yoelii* provided new insights on the remodelling events that occur in non-*falciparum* parasites, including the human parasite *P. vivax.* The molecular events that allow *P. vivax* to remodel its host-cell are currently unknown and require an animal substitute, as *P. vivax* cannot be cultured *in vitro. While P. knowlesi* is phylogenetically closer to *Plasmodium vivax and that the cultivation of P. knowlesi* adapted to human blood was recently described^28^, the remodellome obtained from a lab-adapted parasite is expected to be different from this displayed by parasite grown *in vivo.* Indeed, the exposure to the host is of importance as *Plasmodium* species have developed multiple strategies to evade the host immune response that would be lost upon long-term cultivation. Hence, rodent plasmodium such as *P. yoelii* remains can contribute to provide a first insight on the remodelling events happening in *P. vivax.* Indeed, the fact that *P. vivax* shares the PIR subtelomeric multigene families with rodent malaria parasites suggests that it shares remodelling features with *P. yoelii.* Importantly, our study validated the combined use of subcellular fractionation along with proteomics to identify the parasite remodellome. The high level of functional redundancy observed predicts that the parasite might use alternative proteins/pathways to fulfil remodelling requirements and thus, several targets might be required in order to interfere with this essential aspect of the *Plasmodium* development.

## Materials and Methods

### Ethics statement

This study was carried out in strict accordance with the recommendations of the NACLAR (National Advisory Committee for Laboratory Animal Research) guidelines under the Animal & Birds (Care and Use of Animals for Scientific Purposes) Rules of Singapore. The protocol was approved by the Institutional Animal Care and Use Committee (IACUC) of the Nanyang Technological University of Singapore (Approval number: ARF SBS/NIE-A-0223 AZ). All efforts were made to minimize suffering.

### Fractionation of the parasite samples used for proteomic analysis

BALB/c mice were infected by intraperitoneal injections with *Plasmodium yoelii* 17XNL expressing GFP, obtained from the Malaria Research and Reference Reagent Resource Center (MR4). Highly purified samples of trophozoite and schizont stage parasites were obtained by Nycodenz gradient (Sigma) as previously described^29^. Parasites were divided into two equal aliquots of ~100 μl each. The two aliquots were lysed for 4 min at room temperature in presence of protease inhibitor (Pierce) either with 1600 units of Streptolysin O (Abcam) or with 0.05% of Saponin (Sigma) and separated into pellets and supernatants by centrifugation. Prior to resuspension in SDS sample buffer, the pellet fractions were washed twice using RPMI whereas the supernatant fractions were solubilized immediately. The whole procedure was repeated until four biological replicates were obtained.

### Western-blot analysis of parasite preparations after subcellular fractionation

Western blotting was performed on pellets and supernatants from *infected RBC* lysed with SLO or Saponin. The membranes were probed using chicken anti-GFP sera (Abcam) (GFP), rabbit anti-EXP2 sera (Genscript) (EXP2) recognizing the peptide KNIESGKYEFDVD and rabbit anti-RHAG antibody (Abcam). All the membranes were revealed using alexa 649 or 549 secondary antibody, coupled to a GE typhoon trio scanner.

### Protein Separation, Protein Digestion, Peptide Extraction and LC–MS/MS analysis

As the sub-proteomes contain detergents, proteins from each fractions were separated on 12% SDS-PAGE at 50 V and protein bands were visualized by staining with imperial comassie blue (Pierce). The gel lanes corresponding to each of the fractions (SLO SN, SAPO SN, SLO pellet, SAPO pellet) were cut into 10 separate slices, then de-stained, and the proteins reduced by using dithiothreitol (DTT) and alkylated by iodoacetamide (IAA). The proteins were cleaved by overnight digestion in porcine trypsin (Sequencing Grade Modified, Promega, Wisconsin). The tryptic peptides were extracted by using 5% acetic acid in 50% acetonitrile and vacuum-dried by speedvac. The vacuum concentrated peptides were reconstituted in 0.1% formic acid and analyzed using a LC-MS/MS system. The LC-MS/MS analysis was performed using a LTQFT Ultra mass spectrometer (Thermo-Finnigan, Bremen, Germany), coupled with an online HPLC system (Shimadzu, Kyoto, Japan). The sample (100 μl) from an auto-sampler (Shimadzu, Japan) was injected and concentrated in a Zorbax peptide trap (Agilent, Palo Alto, CA, USA). The peptide separation was performed in a capillary column (200 μm ID × 10 cm) packed with C18 AQ (5 μm, 300 Å pore, Michrom BioResources, CA, USA). The mobile phase A (0.1% formic acid in H_2_O) and B (0.1% formic acid in acetonitrile) were used to establish a 60 min gradient that comprised of 35 min 5–25% B; followed by 10 min 25–60% B; maintained at 80% B for 5 min and finally re-equilibrated at 5% B. The HPLC was maintained at constant flow rate of 30 μL/min and a splitter was used to create a flow rate of approximately 500 nL/min. The samples were injected into LTQFT Ultra through an ADVANCE™ CaptiveSpray™ Source (Michrom BioResources, CA, USA) with an electrospray potential of 1.5 kV. The gas flow was set at 2, ion transfer tube temperature at 180 °C and collision gas pressure at 0.85 mTorr. The LTQFT was set to perform data acquisition in the positive ion mode and full MS scan (350–1600 m/z range) was acquired in the FT-ICR cell at a resolution of 100,000 and a maximum ion accumulation time of 1000 ms. The MS/MS spectra of peptide ion fragments generated by collision-induced dissociation were acquired in the linear ion trap. The 10 most intense ions above a 500 counts threshold were selected for fragmentation in CID.

### Database searching

All MS/MS spectra were searched using in-house Mascot server (version 2.3.2, Matrix Science, Boston, MA, USA). The UniProt mus musculus proteome, the *P. yoelii* 17X proteome in PlasmoDB 13.0 and common contaminant database (http://maxquant.org/contaminants.zip and ftp://ftp.thegpm.org/fasta/cRAP/crap.fasta) were combined and used for the searches. For Mascot search, the raw data files were converted into the mascot generic file format using an in-house program prior to Mascot search, as described previously^30^. In the search, enzyme limits were set at full tryptic cleavage at both ends with a maximum of two missed cleavages and mass tolerances of 10 ppm for peptide precursors. Mass tolerance of 0.8 Da was set for fragment ions in Mascot searches. Estimation of the protein abundance was performed using exponentially modified Protein Abundance Index (emPAI) values reported by Mascot search engine^16^. The emPAI is defined as emPAI = 10^PAI^ – 1 with PAI = *N*_observed_/*N*_observable_ (Eq. 1). *N*_observed_ is the number of experimentally observed peptides and *N*_observable_ is the calculated number of observable peptides for each protein. The emPAI showed here were calculated by merging the data obtained from the four biological replicates. Only proteins identified by at least one unique peptide and found in at least two replicates were retained. EmPAI values were normalized according to the mean value of the total abundance of all protein found in both supernatant and pellet fraction. Finally, the features and the conservation of the proteins presented here were checked using Plasmodb (www.plasmodg.org) (Supplementary Table S1). For PEXEL proteins, only those with an ExportPred Score ≥4.3 were considered as PEXEL positive. The essentiality of the proteins identified was sought by searching in RMgmDB (http://www.pberghei.eu/) and Phenoplasm (http://phenoplasm.org/) databases for syntenic proteins in *P. berghei* and *P. falciparum* for which experimental evidence pertaining to their essentiality obtained using gene deletion approaches, were available. This dataset was completed by reverse genetic data of nine exported candidates found in the PlasmGEM database (http://plasmogem.sanger.ac.uk/)^31^. Overall, One hundred and eighty one syntenic protein with reverse genetic data were classified as “essential” (no gene-deleted parasite obtainable), “requisite” (gene-deleted parasites with a growth defect) or “dispensable” (no phenotype observed).

### Determination of the protein localization using GFP-tagging

BALB/c mice were infected with *Plasmodium yoelii* 17 × 1.1 parasites by intraperitoneal injections. Transfections were carried out as previously described^29^. To express parasite proteins fused to a GFP tag, DNA sequence corresponding to the cognate gene full coding sequence available in PlasmoDB (www.plasmodb.org) were first generated by PCR amplification of genomic DNA/cDNA or chemical synthesis (GeneArt, Genscript or Integrated DNA Technologies). All the CDS were cloned into the plasmid ePL (containing both the *P. berghei* *eef1*-α constitutive promoter, the 3′ UTR region of *P. berghei* DHFR/TS gene and the selectable marker *T. gondii* DHFR gene), upstream of an *egfp*-tag^8^. Infected blood samples obtained after *in vivo* (*P. yoelii*) pyrimethamine selection, were stained with DAPI (1 μg/ml) for 5 min. Transfected parasites expressing GFP-tagged chimeras were then observed with an Olympus IX71 fluorescent microscope using a 100X oil immersion objective. DAPI was detected using a Chromas 11000v3 filter set whereas eGFP was detected using a Chroma 49011 Filter Set. Pictures were captured using an Olympus DP30BW camera and processed using ImageJ 1.42a. Throughout the study, pictures of 20 independent observations of trophozoite/early schizont stage parasites were collected and a representative example showed.

### Characterization of the PYST PY17X_0920900 protein using specific antisera

In addition to GFP-tagging, the localization of the PY17X_0920900 (Figure S4) was further ascertained using a chicken polyclonal sera that recognized a recombinant PY17X_0920900 protein (Genscript). This anti sera was used to ascertain the localization of PY17X_0920900 using 1) a subcellular fractionation approach that allows us to extract RBC membrane fraction and 2) immunofluorescence on fixed infected RBC. Trophozoite-schizont samples purified using nycodenz gradient were fractionated in presence of protease inhibitor by sequential lysis using SLO and Saponin followed by differential centrifugation^32^. Briefly, ~50 μl of infected RBC was first treated with 800U of SLO (abcam) for 4 min at RT. The SLO-treated suspension was then centrifuged at 800 *g* for 5 min to separate the parasite within the PVM (pellet) from RBC cytoplasm and the RBC membrane (supernatant). Following three additional washes using iRPMI, the pellet was then treated with 0.05% of Saponin and centrifuged to separate PV/PVM component (supernatant) from the intraerythrocyte parasite (pellet). Following three additional washes, the pellet fraction that contains internal parasite was dissolved in Laemmli sample buffer. SLO and Saponin supernatants were clarified by centrifugation at 20,000 *g* for 40 min to isolate the RBC membrane or the PVM (PVM 1), respectively. Both RBC membrane and PVM 1 pellets were washed 4 times by resuspension in RPMI and centrifuged at 20,000 *g* for 40 min. Following the first centrifugation at 20,000 g, the resulting SLO and Saponin supernatants were ultracentrifuged at 300,000 *g* for 1 hour to separate the specialized structure (HCC vesicles) from the soluble content of the HCC (HCC soluble proteins) and the PVM content that was not isolated by the first centrifugation (PVM 2) from the PV content (PV). In total, six fractions were produced and dissolved in an equivalent amount of Laemmli sample buffer: RBC membrane, HCC vesicles, HCC soluble protein, PVM 1, PVM 2, PV and internal parasite. Parasite equivalents to 5 μl of infected RBC pellet were loaded on protein gel and analysed by Western blot with anti- PY17X_0920900 sera. The membrane probed with anti- PY17X_0920900 sera was revealed using a cognate alexa 649 secondary antibody coupled to a GE typhoon trio scanner capture.

To study the localizations of the proteins, air-dried *P. yoelii* infected erythrocyte fixed for 5 minutes with PFA (Pierce) were first stained with specific anti-serum (dilution 1/200) in conjunction with anti-chicken secondary antibody coupled to Alexa Fluor 488 (Invitrogen). The parasite PVM was then revealed using rabbit anti-EXP2 antibody in conjunction with anti-rabbit secondary antibody coupled to Alexa Fluor 594 (Invitrogen). Non-immune mouse serum was used as a control. Slides were then examined by fluorescence microscopy after a brief incubation with DAPI (2 μg/ml).

## Additional Information

**How to cite this article**: Siau, A. *et al.* Proteome mapping of Plasmodium: identification of the *P. yoelii* remodellome. *Sci. Rep.* **6**, 31055; doi: 10.1038/srep31055 (2016).

## Supplementary Material

Supplementary Table S1

Supplementary Information
