# Corrigendum: Proteome mapping of *Plasmodium*: identification of the *P. yoelii* remodellome

**DOI:** 10.1038/srep37202

**Published:** 2016-11-21

**Authors:** Anthony Siau, Ximei Huang, Mei Weng, Siu Kwan Sze, Peter R. Preiser

Scientific Reports 6: Article number: 31055; 10.1038/srep31055 published online: 08 09 2016; updated: 11 21 2016.

The Acknowledgements section in this Article is incomplete.

“The authors are grateful to Ms A. van Wigcheren and Dr. C.J. Jance for their protein localization data, Dr. O. Billker and Dr. J.C. Rayner for their reverse genetic information and Dr. O. Sheriff and Mr. D.W.J. Tay for the critical reading of the manuscript. This work was supported by the National Medical Research Council (Singapore) (NMRC/CBRG/0040/2013)”.

should read:

“The authors are grateful to Ms A. van Wigcheren and Dr. C.J. Jance for their protein localization data, Dr. O. Billker and Dr. J.C. Rayner for their reverse genetic information and Dr. O. Sheriff and Mr. D.W.J. Tay for the critical reading of the manuscript. This research is supported by the Singapore Ministry of Health’s National Medical Research Council under its Cooperative Basic Research Grant (NMRC/CBRG/0040/2013), and Singapore Ministry of Education Academic Research Fund Tier 2 (MOE2012-T2-2-093)”.

