# Supplementary material for: Proteome mapping of Plasmodium: identification of the P. yoelii remodellome
Source: Sci Rep. 2016 Aug 9;6:31055. doi: 10.1038/srep31055 (PMC4977464; doi:10.1038/srep31055)
Supplement: Supplementary Information [file srep31055-s2.doc]

***Proteome mapping of Plasmodium: identification of the P. yoelii remodellome***

Anthony Siau, Ximei Huang, Mei weng*,* Siu Kwan Sze and Peter R Preiser

**Supplementary Figures**

**Figure S1**


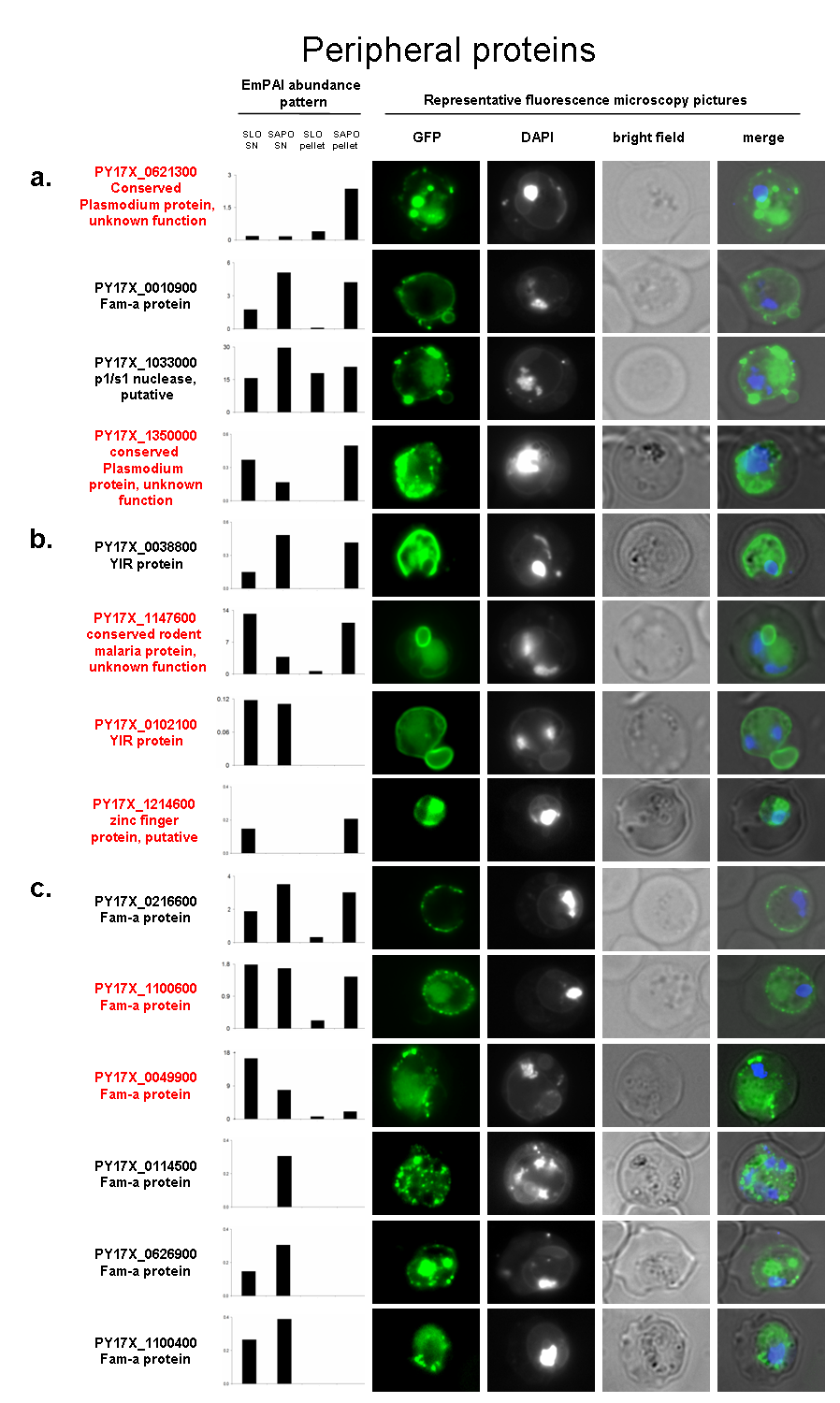


**Figure S1.** **Representative pictures of parasites expressing peripheral proteins fused to a GFP-tag.** Left panel shows the protein ID and the cognate description available in PlasmoDB database. Middle panel shows the detailed relative abundance pattern found by proteomic analysis. Right panel shows the GFP fluorescence pattern of these peripheral proteins in live *P. yoelii* infected erythrocyte as determined by fluorescence microscopy of fluorescently-tagged proteins. The proteins were grouped based on their GFP fluorescence patterns. **a.** punctate and smooth pattern **b.** smooth GFP pattern. **c.** punctate pattern. The peripheral proteins mispredicted as exported or internal by proteomic data were highlighted in red. Red arrows indicate a circular compartment in the parasite or the HCC of PY17X_1147600 and PY17X_0102100 transfectants, that could be related to the central cavity recently described in blood stage parasites (Gruring, C. *et al.* 2011*)*. The blue arrow shows whorl-like extension(s) emerging from the parasite body of a subset of transfectants, which could be related to the *P. falciparum* TVN (Lauer, S.A. *et al.* 1997). The pattern observed for the nuclease PY17X_1033000 transfectants overlapped with that observed for EXP2, with fluorescence localized into whorl-like extensions and in some of the connection points between the whorl-like extension and the parasite’s main body as highlighted by the yellow arrow (Riglar, D.T. *et al.* 2013).

**Figure S2**


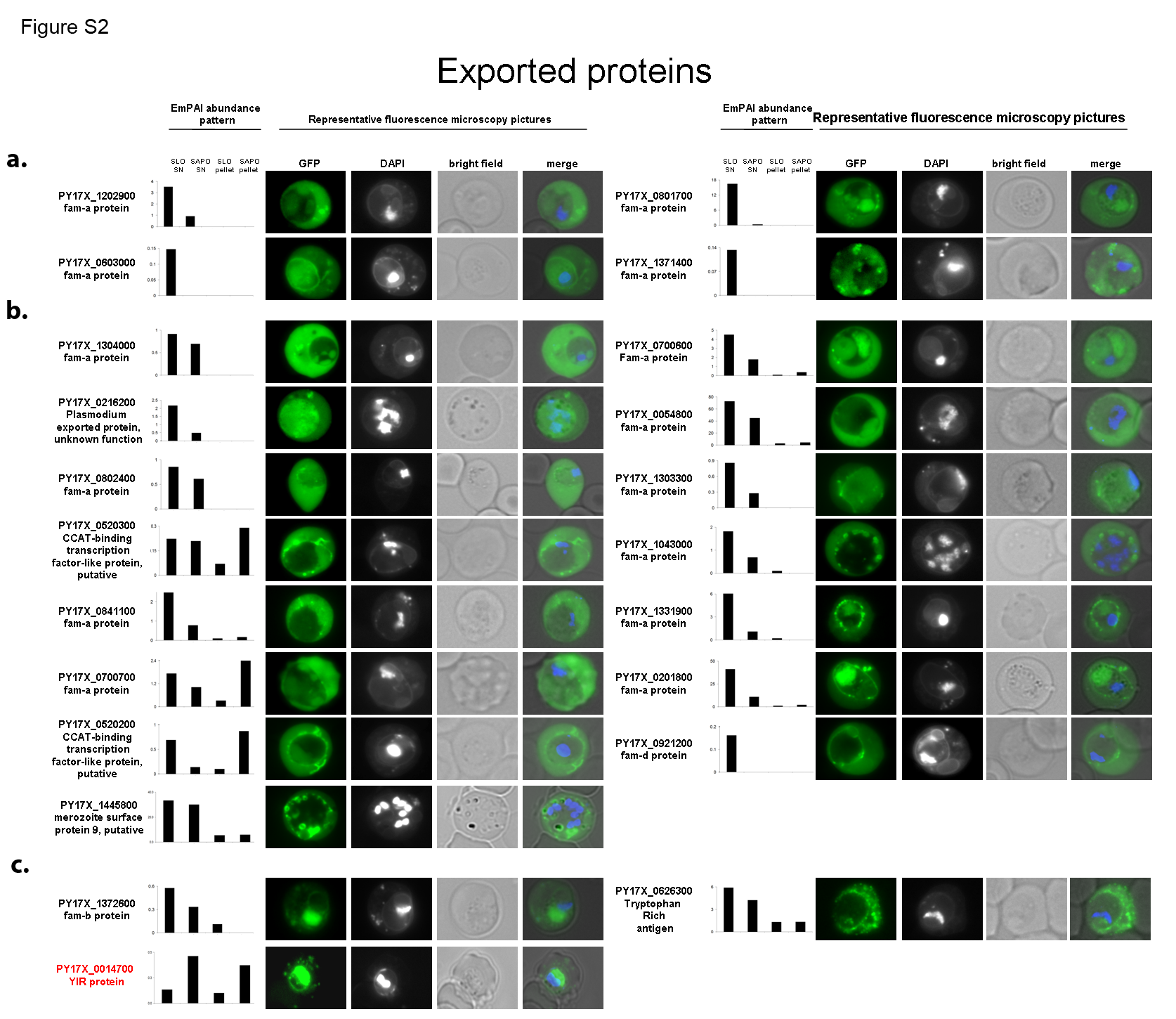


**Figure S2.** **Representative pictures of parasites expressing exported proteins fused to a GFP tag.** For each protein (left panel), the description available in PlasmoDB database is indicated below the protein ID while the corresponding relative abundance pattern and the representative photographs of *P. yoelii* infected erythrocyte as determined by fluorescence microscopy of fluorescently-tagged proteins are represented in the middle and right panels, respectively. The proteins are grouped according to their GFP fluorescence patterns; **a.** punctate and smooth pattern **b.** smooth GFP pattern. **c.** punctate pattern. The exported protein mispredicted as peripheral by proteomic data were highlighted in red..

**Figure S3**


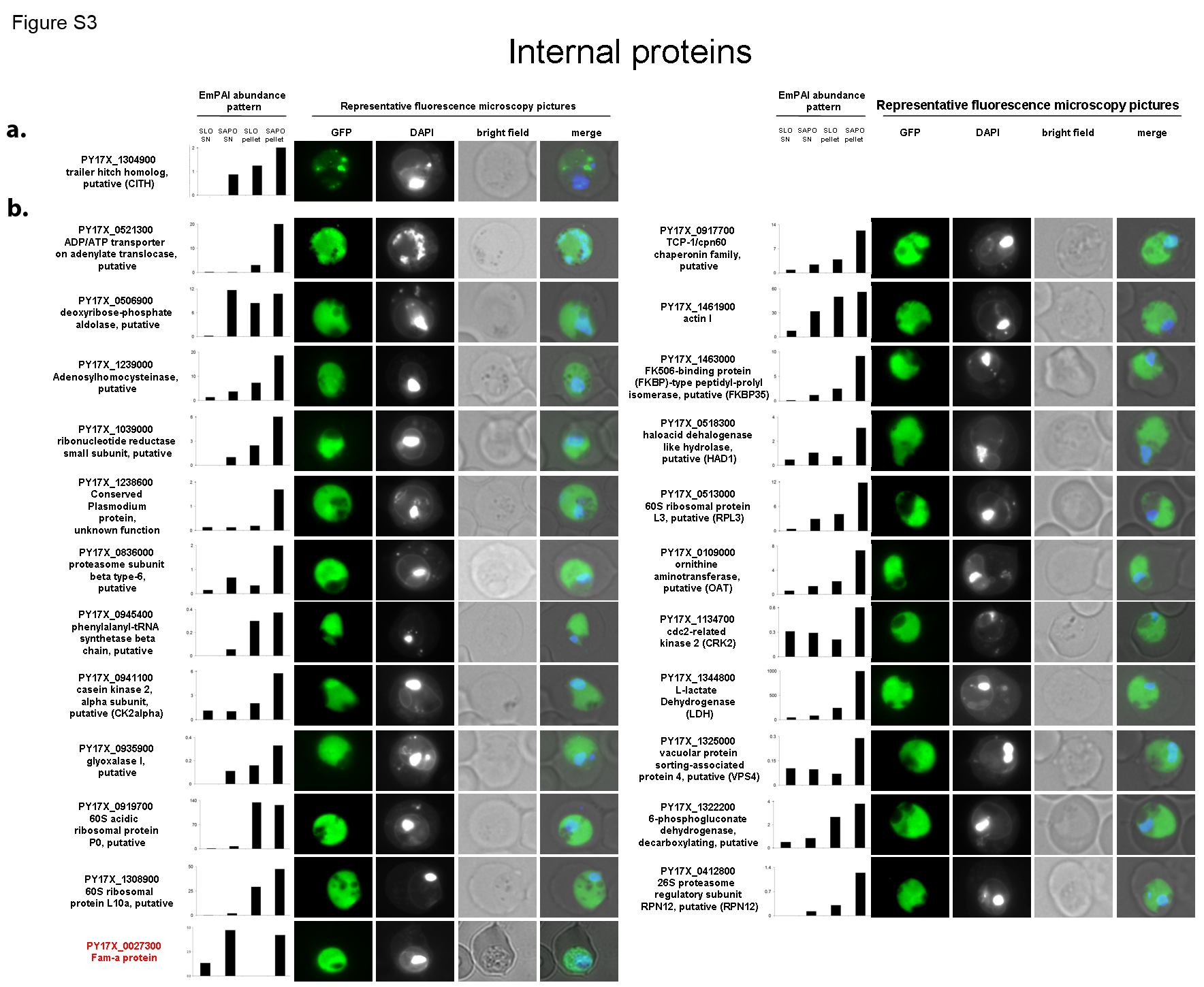


**Figure S3.** **Representative pictures of parasites expressing internal proteins fused to a GFP tag.** Localization of internal proteins in live *P. yoelii* infected erythrocyte, as determined by fluorescence microscopy of fluorescently-tagged proteins. For each protein, the description available in PlasmoDB database is indicated below the protein ID while the corresponding relative abundance pattern and the representative photographs of infected erythrocytes are represented in the middle and right panels, respectively. The proteins are grouped according to their GFP fluorescence pattern; **a.** punctate and smooth pattern **b.** smooth GFP pattern. The internal protein mispredicted as peripheral by proteomic data were highlighted in red..

**Figure S4**


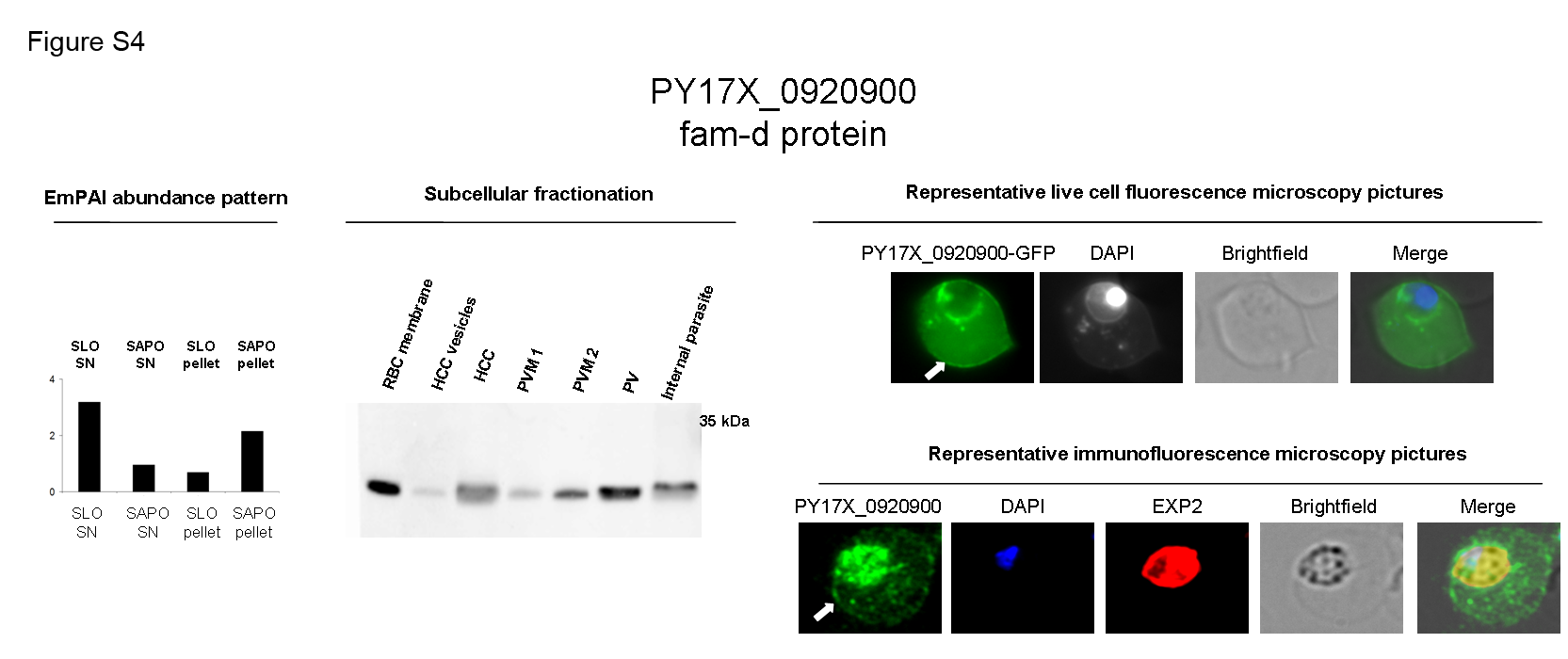


**Figure S4. Characterization of PY17X_0920900, a PYST associated to RBC membrane**. Left panel shows the relative abundance pattern found by proteomic analysis for PY17X_0920900. Middle panel shows the abundance of the protein in multiple subcellular compartments isolated using a sub-cellular approach based on sequential treatment of wild type infected RBC with SLO and Saponin (see materials and methods). Each lane was loaded with ≈5 µl of equivalent parasites. Western blot, using antibodies against the PY17X_0920900, was used to evaluate the protein abundance. The molecular weight on the right indicates the positions to which size markers had migrated. Right panel display the representative images of *P. yoelii* infected erythrocyte expressing full-length PYST fused to GFP, as determined by live cell microscopy (top) or immunofluorescently labelled proteins on fixed infected RBC (bottom). The RBC membrane is indicated by a white arrow.

Out of the 63 proteins screened here, only transfectants expressing the exported PYST PY17X_0920900 protein (771 bp ORF encoding a 30kDa polypeptide with a signal peptide) displayed a circular fluorescence that overlaps with the membranes revealed by DAPI labelling, suggesting that a part of this protein could be associated with the RBC membrane (right top). To confirm this*, P. yoelii* infected RBC were subjected to sequential lysis of the different membranes surrounding the parasite using SLO and Saponin in order to separate the different extra-parasite compartments of the infected RBC (Shastri, S. *et al.* 2010). Polyclonal antibody generated using the PY17X_0920900 recombinant protein revealed a band of ~28kDa in all the fractions, including those enriched in RBC ghosts (middle). Similarly, IFA performed on fixed infected RBC displayed a circular fluorescence external to the PVM labelling, as revealed by anti EXP-2 antibody (bottom right). However, the repeated absence of staining noted when live WT *P. yoelii* were labelled with anti-PY17X_0920900 antibody (data not shown), suggests that PY17X_0920900 protein found at the RBC membrane is likely exposed to the cytoplasmic side of the membrane or beneath it.
